# Liver Regeneration after Partial Hepatectomy Is Not Impaired in Mice with Double Deficiency of *Myd88* and *IFNAR* Genes

**DOI:** 10.1155/2011/727403

**Published:** 2011-12-19

**Authors:** Javier Vaquero, Kimberly J. Riehle, Nelson Fausto, Jean S. Campbell

**Affiliations:** ^1^Department of Pathology, University of Washington, Seattle, WA 98195, USA; ^2^Laboratorio de Investigación en Hepatología y Gastroenterología, HGU Gregorio Marañón-CIBERehd, 28009 Madrid, Spain; ^3^Department of Surgery, University of Washington, Seattle, WA 98195, USA

## Abstract

Liver regeneration is known to occur in mice lacking one or more Toll-like receptors (TLRs) or the adaptor protein MyD88. Though MyD88 is required for signaling by many TLRs, others signal via MyD88-independent pathways, leading to the induction of type I interferons (IFNs). Here, we assessed liver regeneration after partial hepatectomy (PH) in mice lacking both MyD88 and the type I IFN receptor (*Myd88-IFNAR* double-KO). Approximately 28% of *Myd88-IFNAR* double-KO mice had gross liver lesions prior to surgery. In mice without lesions, *Myd88-IFNAR* deficiency abrogated the increase in circulating IL-6 after PH but did not impair hepatocyte BrdU incorporation, mitotic figure counts, or recovery of liver-to-body weight ratios. These results indicate that type I IFNs are not responsible for the preservation of liver regeneration in *Myd88*-deficient mice, and they also cast doubt on the idea of microbial products being essential triggers of liver regeneration in mice undergoing PH.

## 1. Introduction

Liver regeneration after partial hepatectomy (PH) depends on the ability of hepatocytes and nonparenchymal cells (NPCs) to rapidly integrate multiple signals originating from immune, hormonal, and metabolic networks [1, 2]. A consequence of such integration is the induction of proinflammatory cytokines (tumor-necrosis-factor- (TNF-) *α*, interleukin- (IL-) 6) in the liver, most likely in resident macrophages (Kupffer cells) [3–5]. In mice, this cytokine activation results in an early increase of IL-6 in the circulation, which modulates a variety of genes involved in cell proliferation, survival, and the acute phase response [6, 7].

Early studies performed in germ-free, lipopolysaccharide- (LPS-) resistant, and antibiotic-treated rodents suggested that microbial products from intestinal bacteria reaching the remnant liver in relative excess are responsible for triggering liver regeneration after PH [8, 9]. It is now known that the cellular detection of microbial components mostly relies on the recognition of pathogen-associated molecular patterns (PAMPs) by the evolutionarily conserved Toll-like (TLR)/IL-1 family of receptors, although non-TLR pathways also exist [10, 11]. In accordance with the notion that intestinal bacteria trigger liver regeneration, several studies have consistently shown an abrogation of circulating IL-6 after 2/3 PH in mice deficient in MyD88, an adaptor protein for all TLR/IL-1 receptors except TLR-3 [12–14]. In a detailed assessment of mice lacking one or more TLR genes, we recently reported that up to 60% of the increase in IL-6 after PH depends on signaling by the LPS receptor TLR-4, but other MyD88-dependent ligands/receptors contributing to IL-6 production could not be identified [15]. Despite the contributions of TLR-4 and MyD88 to IL-6 production after PH, full restitution of liver mass after PH was observed in mice lacking one or more TLRs, including *Tlr4* KO, *Tlr2* KO, *Tlr9* KO, *Tlr2,4* double-KO, *Tlr2,4,9* triple-KO, and *Tlr2,4-caspase-1* triple-KO mice, as well as in *Myd88* KO mice [12, 13, 15]. Whereas the deficiency of *Myd88* resulted in an earlier initiation of hepatocyte proliferation under our experimental conditions [15], Seki et al. found a transient impairment of hepatocyte proliferation in the same mouse strain [12]. Remarkably, all studies showed that *Myd88*-null mice regenerated their livers to the same extent as littermate controls, despite the profound defect in IL-6 production. In a separate study, a transient acceleration of hepatocyte proliferation after PH was described in mice deficient in *Tlr3* [16], a TLR that signals via a MyD88-independent pathway resulting in the induction of interferon- (IFN-) *α* and *β*. These IFNs, which may also be induced by TLR-4 in a MyD88-independent manner, signal via the type I IFN receptor (IFNAR), and characteristically result in the induction of proinflammatory cytokines and chemokines with potent antiviral, antibacterial, and antitumoral properties [17].

A potential explanation for the mild effects of TLR and MyD88 deficiencies on murine liver regeneration may be the great degree of redundancy and cooperation that exist between TLRs and among other non-TLR pattern-recognition receptors [11]. It is conceivable that, after PH, multiple microbial products (lipoproteins, LPS, DNA, etc.) may simultaneously activate different TLRs in the same and/or in different cells. In addition, TLR signaling via MyD88-independent pathways could still occur in *Myd88*-deficient mice. Here, we evaluated liver regeneration in mice with simultaneous deficiency of *Myd88* and *IFNAR* genes to provide further insight into these possibilities. We report that liver regeneration after PH is preserved in *Myd88-IFNAR* double-KO mice, indicating that type I IFNs are not responsible for the preservation of liver regeneration in *Myd88*-deficient mice. Our results do not provide support to the notion of microbial products from intestinal bacteria being essential for murine liver regeneration after PH.

## 2. Material and Methods

### 2.1. Mice

*Myd88-IFNAR* double-knockout (KO) mice were provided by Dr. C. Wilson (Department of Immunology, University of Washington, Seattle, WA) and bred in such a way that mice remained homozygous for the defective alleles. C57Bl6/J wild-type (WT) mice were obtained from The Jackson Lab and bred in parallel with the* Myd88-IFNAR* double-KO mice. All mice were housed in the same room in a specific pathogen-free facility with 12 h light/dark cycles and fed *ad libitum*.

*Myd88-IFNAR* double-KO mice were difficult to breed, often having a lower number of litters per breeding pair, smaller litter sizes, and a higher tendency to have runted mice than their WT controls. Early deaths also appeared to be more common in *Myd88-IFNAR* double-KO mice; these deaths were often (but not exclusively) around the time of parturition in breeding females. The Institutional Animal Care and Use Committee at the University of Washington approved all studies.

### 2.2. Surgeries

Eight-to-twelve week-old male mice (weighing 20–25 grams) underwent 2/3 PH or sham laparotomy under inhalational isofluorane anesthesia (*n* = 3–13 per genotype per time point). 2/3 PH consisted of a midline laparotomy with removal of the left and anterior (median) liver lobes and the gallbladder, as described in [18]. The sham laparotomy group consisted in gentle manipulation of liver lobes without removal of liver tissue, following thereafter the same assessments as mice undergoing 2/3 PH. Surgeries were performed between 7 and 11:30 AM in overnight-fasted mice, and all mice received *∼*0.7 mL of intraperitoneal (IP) sterile 0.9% saline before closing the abdomen, to correct for fluid losses. Mice rapidly regained consciousness and had immediate access to food and water. They were euthanized at indicated times by CO_2_ inhalation, with administration of BrdU (50 mg/kg IP) 2 h before sacrifice. The development of small areas of hepatocyte necrosis was observed in a small percentage of mice of each genotype undergoing PH; these mice were excluded from all analyses. Liver tissue was either immersed in fixative or snap-frozen and stored at −80°C.

### 2.3. Retroorbital Bleeding Technique

A retroorbital bleeding sample (150–200 *μ*L) was obtained 4 h after surgery. For this procedure, mice were reanesthetized with isofluorane, and blood was obtained via a heparinized Natelson capillary tube (Fisher Scientific, Pittsburgh, PA) inserted via the medial canthus approach. The whole procedure from induction to recovery from anesthesia lasted approximately 5 minutes. Blood was centrifuged and plasma stored at –80°C.

### 2.4. Histology and Immunostaining

Liver and small intestine were fixed for 24 h in Methacarn (60% methanol, 30% chloroform, and 10% acetic acid: v/v/v), embedded in paraffin, and cut in 6 *μ*m thick sections. BrdU immunostaining was performed with a monoclonal anti-BrdU antibody (Dako Corp, Carpinteria, CA) as described in [13]. Hepatocyte proliferation was measured by the mean number of hepatocytes with positive BrdU nuclear staining counted in eight 200× microscopic fields (approximately 3,500 hepatocytes) and the total number of mitotic figures in hepatocytes counted in an equivalent area of adjacent, hematoxylin and eosin-stained tissue. Proliferation of nonparenchymal cells (NPCs) was assessed by the mean number of BrdU-labeled NPCs counted in the same area as BrdU-labeled hepatocytes.

### 2.5. Determination of Circulating Cytokines in Plasma

We measured the circulating concentration of IL-6 using a mouse IL-6 ELISA kit (555240, BD Biosciences). We also measured the concentrations of IL-6, TNF-*α*, IL-1*β*, IL-12 p70, IL-10, IFN-*γ*, and VEGF using a bead-based cytometric immunoassay system (Luminex, Austin, TX) according to the manufacturer's instructions (R&D Systems, Minneapolis, MN).

### 2.6. Statistics

Data are presented as mean ± SEM. Differences were analyzed using *U*-Mann Whitney test or the Kruskal Wallis test followed by Dunn's test. A *P* value < 0.05 was considered significant. Analyses were performed using Prism 5.02 (GraphPad Software, Inc.).

## 3. Results

### 3.1. General Characteristics and Presence of Baseline Liver Pathology in *Myd88-IFNAR* Double-KO Mice

**Figure 1:**
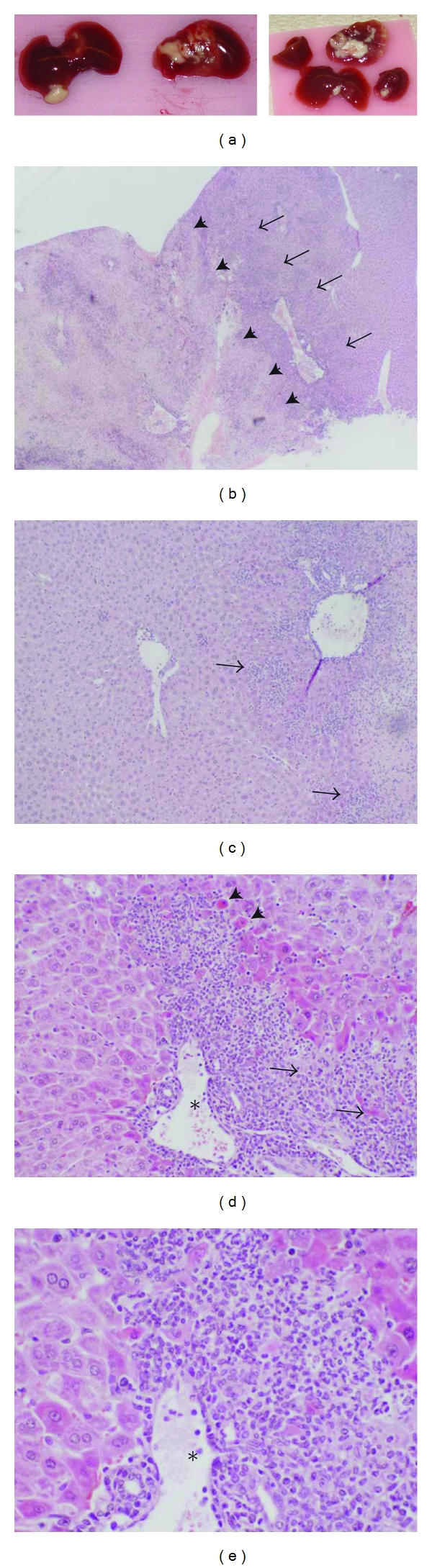
Baseline liver lesions in *Myd88-INFAR* double-KO mice. (a) White lesions on the surface of multiple liver lobes from two male *Myd88-IFNAR* double-KO mice (8 weeks old), detected at the time of laparotomy. Unaffected areas of the liver have a normal appearance. Paraffin-embedded liver tissue sections (6 *μ*m) of abnormal livers were stained with hematoxylin and eosin and digitally captured at 40x (b), 100x (c), 200x (d), and 400x (e) magnification. (b) Large areas of hepatocellular necrosis across the liver lobes (indicated by arrowheads) are surrounded by layers of hepatocytes with eosinophilic cytoplasm and dense inflammatory cell infiltrates (indicated by arrows). Adjacent liver tissue within the same lobe looks normal. (c) Layers of hepatocytes with eosinophilic cytoplasm in zones I and II of the hepatic lobule, with dense inflammatory cell infiltrates (arrows). (d) and (e) Details of zone I of a hepatic acinus showing a dense inflammatory reaction composed of lymphocytic and polymorphonuclear cells. The inflammatory infiltrate encloses hepatocytes undergoing coagulative necrosis (arrows) and a portal vein with histological features of endothelitis (asterisk). The hepatocytes that surround the inflammatory infiltration have an altered cell shape, increased eosinophilia, and some show pyknotic nuclei suggestive of apoptosis (arrowheads).

For the present study, we performed 2/3 PH or sham laparotomy in 8–12-week-old male C57Bl6 WT and *Myd88-IFNAR* double-KO mice. No obvious external physical anomalies were apparent in any of the mice prior to surgery. Unexpectedly, at the time of laparotomy we found that a considerable proportion of *Myd88-IFNAR* double-KO mice had gross liver abnormalities. Specifically, 7 of 25 (28%) *Myd88-IFNAR* double-KO mice had macroscopically visible white lesions on the liver surface, mostly on the median and left lateral lobes (Figure 1(a)). These discolored areas were well demarcated and firmer than the rest of the liver, and surrounding tissues had often adhered to them. Microscopic examination of these lesions revealed large areas of coagulative necrosis (Figure 1(b)). The parenchyma surrounding the necrotic tissue is composed of hepatocytes with increased eosinophilia; some of these cells also have rounded morphology and pyknotic nuclei suggestive of apoptosis. Dense foci of lymphocytic and polymorphonuclear cell infiltrates are particularly prominent in the portal and periportal areas of the hepatic lobule, and they encircle hepatocytes undergoing necrosis. Portal veins with histological features suggestive of endothelitis are also seen (Figures 1(c)–1(e)). The parenchyma surrounding the affected areas as well as the parenchyma of nonaffected lobes is remarkably normal.

Only *Myd88-IFNAR* double-KO mice that did not have intraoperative liver pathology were included in the primary analyses. Body weight was similar in WT and *Myd88-IFNAR* double-KO mice at the time of surgery (21.2 ± 0.5 g versus 21.1 ± 0.6 g, NS), and they also gained similar body weight during the first 7 days after the PH (data not shown), supporting the absence of serious illness in the *Myd88-IFNAR* double-KO mice included in the analyses.

### 3.2. Deficit in Circulating IL-6 Levels after PH in *Myd88-IFNAR* Double-KO Mice

**Figure 2:**
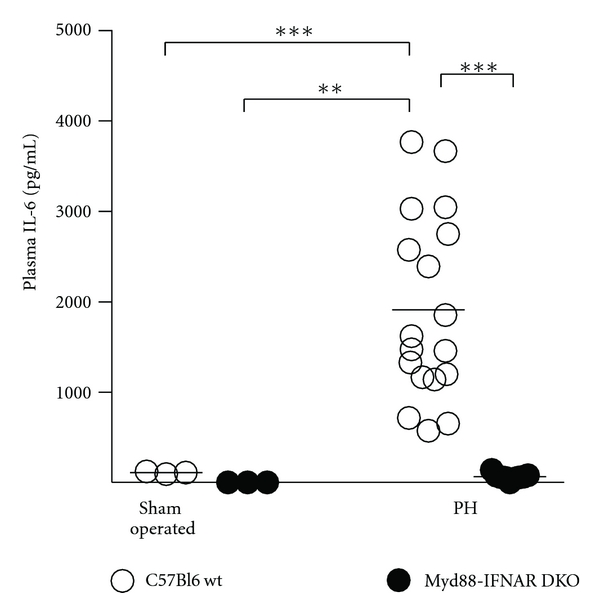
Circulating levels of IL-6 in C57Bl6 WT mice and *Myd88-IFNAR* double-KO mice after PH or sham laparotomy. Blood samples were obtained from the retroorbital sinus at 4 h after surgery, and IL-6 was measured in plasma by ELISA. **P < 0.01, ***P < 0.001.

After PH, there is an early increase of circulating IL-6 that has been shown to depend on signaling through MyD88. In the present study, we measured circulating levels of IL-6 by ELISA in retroorbital bleeding samples obtained 4 h after sham operation or PH. Circulating IL-6 is undetectable in nonoperated WT mice (data not shown) and in sham-operated *Myd88-IFNAR* double-KO mice (Figure 2). Plasma IL-6 markedly increases in WT mice after PH, compared with a slight elevation observed after sham laparotomy. The increase of circulating IL-6 after PH is profoundly abrogated in *Myd88-IFNAR* double-KO mice, which have IL-6 levels equivalent to those measured in sham-operated mice.

We then used the Luminex platform to assess whether the double deficiency of *Myd88* and *IFNAR* is associated with changes in other circulating cytokines after PH. While the Luminex assay confirms the changes in IL-6 that we had measured by ELISA (WT + sham: 193 ± 23, WT + PH: 1732 ± 274, *Myd88-IFNAR* double-KO + PH: 139 ± 43 pg/mL, *P* < 0.01 WT + PH versus the rest), the circulating concentrations of TNF-*α*, IL-1*β*, IL-12 p70, IL-10, and IFN-*γ* are below the range of detection of the assay in the vast majority of WT and *Myd88-IFNAR* double-KO mice after sham operation, as well as after PH. Circulating levels of VEGF are detectable in sham-operated WT mice and they tend to decrease to a similar extent after PH in both WT and *Myd88-IFNAR* double-KO mice (WT + sham: 94.5 ± 27.8, WT + PH: 30.3 ± 2.0, *Myd88-IFNAR* double-KO + PH: 34.8 ± 2.0 pg/mL, *P* = 0.054).

### 3.3. Preserved Liver Regeneration in *Myd88-IFNAR* Double-KO Mice without Preexisting Liver Pathology

All *Myd88-IFNAR* double-KO mice without preexisting liver pathology that underwent PH survived, and they do not have increased rates of postoperative hepatocellular necrosis compared with WT mice. The ratios of the weight of the resected liver lobules in relation to the body weight were similar in WT mice and *Myd88-IFNAR* double-KO mice undergoing PH (2.40 ± 0.06% versus 2.31 ± 0.07%, NS), supporting the absence of gross differences in the extent of tissue resection or liver mass at baseline.

**Figure 3:**
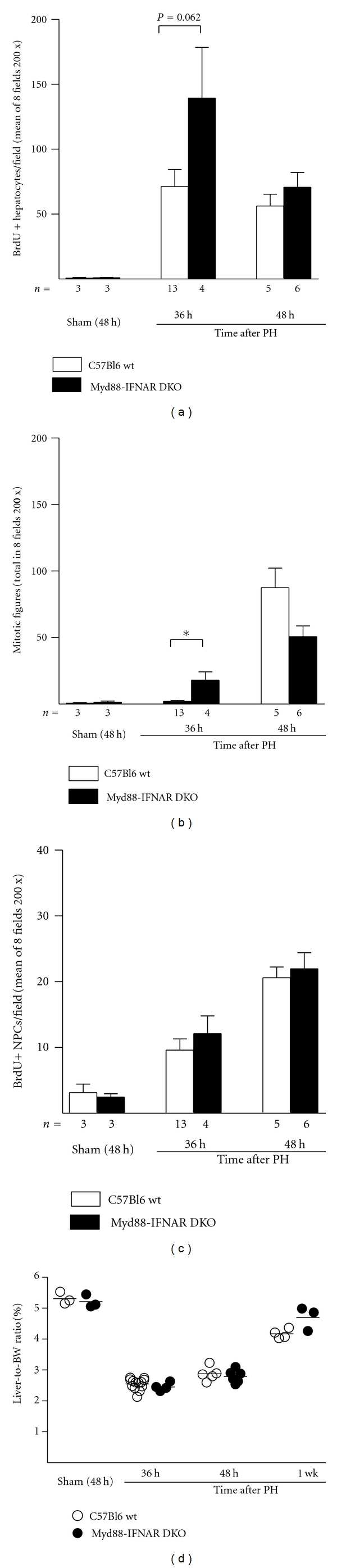
Liver regeneration after PH in C57Bl6 WT and *Myd88-IFNAR* double-KO mice without preexisting liver lesions. Shown are (a) the mean number of BrdU-labeled hepatocytes, (b) the total number of mitotic figures in hepatocytes, and (c) the mean number of BrdU-labeled nonparenchymal liver cells (NPCs), counted in eight 200x microscopic fields per mouse liver in C57Bl6 WT mice (white bars) and in *Myd88-IFNAR* double-KO mice (black bars) at the indicated times after PH or sham laparotomy. The number of mice in each group is indicated below the *x*-axis. *P < 0.05. (d) Liver-to-body weight ratios in C57Bl6 WT (white circles) and *Myd88-IFNAR* double-KO mice (black circles), measured at the indicated time points after PH or sham laparotomy.

We evaluated the extent of hepatocyte proliferation after PH or sham laparotomy by counting the number of BrdU-labeled hepatocytes and mitotic figures in paraffin-embedded liver tissue sections. As shown in Figures 3(a) and 3(b), hepatocyte proliferation is minimal in healthy WT and *Myd88-IFNAR* double-KO mice undergoing sham operations. The number of BrdU-labeled hepatocytes significantly increases after PH in both strains of mice, with a nonsignificant trend (*P* = 0.062) towards increased hepatocyte BrdU labeling at 36 h after PH in *Myd88-IFNAR* double-KO mice compared with WT mice (Figure 3(a)). The number of mitotic figures also increases after PH in both groups (Figure 3(b)) and is significantly higher in *Myd88-IFNAR* double-KO mice compared with WT controls at 36 h after PH (*P* = 0.02). No significant differences between genotypes are observed at the peak of mitosis (48 h after PH), however. BrdU labeling of NPCs also increases after PH in both WT and *Myd88-IFNAR* double-KO mice, but no significant differences between strains were noted at any time point (Figure 3(c)). Both groups of mice have similar liver-to-body weight ratios 48 h after sham laparotomy, as well as remarkably similar liver-to-body weight ratios at 36 h, 48 h, and 7 days after PH (Figure 3(d)).

### 3.4. Abnormal Liver Regeneration in *Myd88-IFNAR* Double-KO Mice with Preexisting Liver Pathology

**Figure 4:**
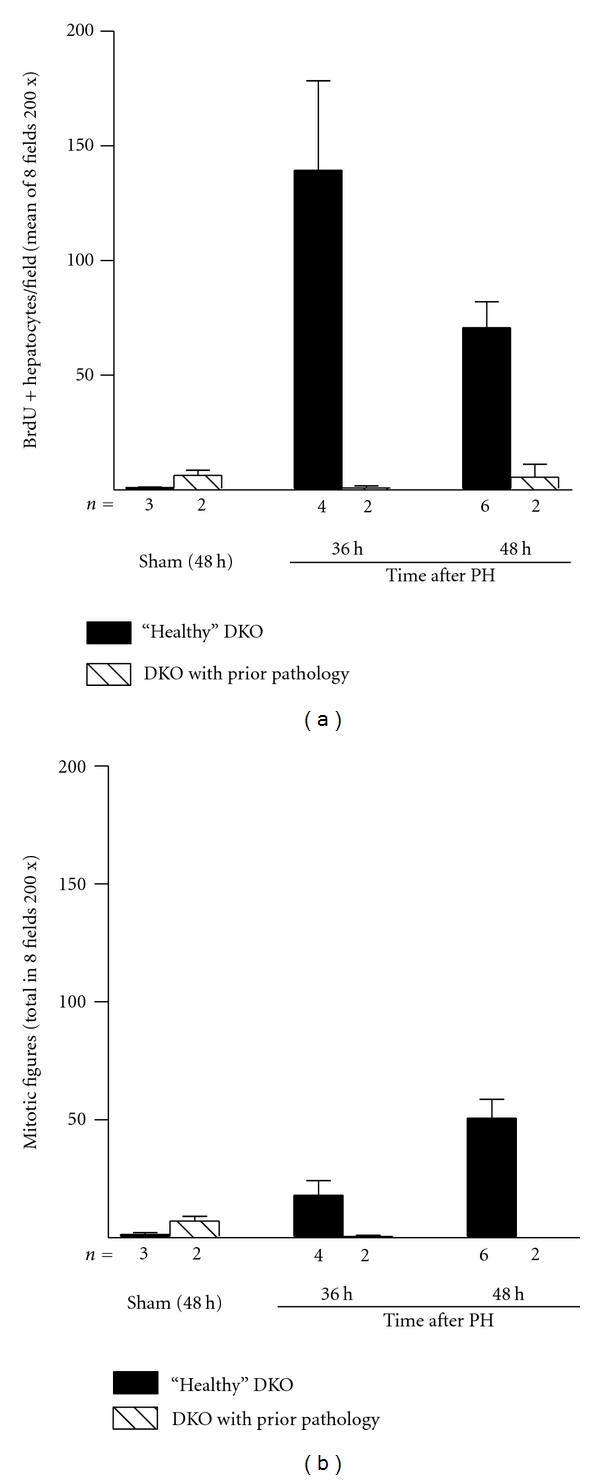
Impact of preexisting liver lesions on hepatocyte proliferation after partial hepatectomy (PH) in *Myd88-IFNAR* double-KO mice. The graphs show (a) the mean number of BrdU-labeled hepatocytes and (b) the total number of mitotic figures in hepatocytes, counted in eight 200x microscopic fields per mouse liver at the indicated times after PH or sham laparotomy in *Myd88-IFNAR* double-KO mice without (black bars) and with (hatched bars) preexisting liver lesions. The number of mice in each group is indicated below the *x*-axis. The dataset for *Myd88-IFNAR* double-KO mice without preexisting liver lesions is the same as that shown in Figure 3. The small sample size precludes a formal statistical analysis.

In mice undergoing sham operations, the number of BrdU-positive hepatocytes and mitotic figures tends to be higher in* Myd88-IFNAR* double-KO mice with preexisting liver lesions compared with those with grossly normal livers, perhaps as a reflection of ongoing liver injury (Figures 4(a) and 4(b)). *Myd88-IFNAR* double-KO mice with preexisting liver pathology, however, seem to have impaired hepatocyte proliferation after PH compared with the “healthy” *Myd88-IFNAR* double-KO group, both by the number of BrdU-positive hepatocytes and mitotic figures (Figures 4(a) and 4(b)). The low number of cases at each time point impedes formal statistical comparisons between these two groups. Noteworthy, the resection of liver lobules in some mice with preexisting liver lesions was technically challenging, due to the presence of adhesions between the necrotic areas of the liver and surrounding tissues.

## 4. Discussion

The present study was performed to investigate Cornell's original hypothesis that intestinal microorganisms are essential triggers of cytokine activation and liver regeneration after PH [8, 9]. The main finding of the present study is that liver regeneration after PH is not impaired by the simultaneous deficiency of *Myd88* and *IFNAR* genes. The *Myd88-IFNAR* double-KO paradigm leads to extensive defects in TLR signaling, as it disrupts all MyD88-dependent pathways as well as signaling by type I IFNs, which are major mediators induced by TLRs via MyD88-independent pathways [11]. Notably, previous studies have shown that the response of the innate immune system to pathogens is more impaired in *Myd88-IFNAR* double-KO mice than in mice with single deficiencies of these genes [19–21].

In recent studies, we and others have shown that MyD88, an adaptor protein for many TLRs and the IL-1R, is required for cytokine activation after PH [12, 13] and that signaling via the LPS receptor TLR-4 contributes to up to 60% of the increase in IL-6 after PH [15]. Liver regeneration, however, is preserved in *Tlr4* KO, *Tlr2* KO, *Tlr9* KO, *Tlr2,4* double-KO, *Tlr2,4,9* triple-KO, *Tlr2,4-caspase-1* triple-KO mice, and, most surprisingly, in mice lacking *Myd88*. Though these findings suggested that TLR ligands and MyD88 are not essential for liver regeneration, redundancy among TLRs and signaling downstream of TLRs via MyD88-independent pathways were still possible [11, 17]. MyD88-independent pathways downstream of TLR-3 and TLR-4 consecutively involve the adaptor protein TIR-domain-containing adaptor-inducer interferon-*β* (TRIF), the activation of interferon regulatory factor-3 (IRF-3), and the subsequent induction of type I IFNs and other genes. In the present study, the additional absence of the type I IFN receptor in *Myd88-IFNAR* double-KO mice did not uncover any substantial contributions of this MyD88-independent pathway to normal liver regeneration. Similar to our previous study in *Myd88* KO mice [15], we even noted a trend towards an accelerated initiation of hepatocyte proliferation in *Myd88-IFNAR* double-KO mice. It should be noted that TLR signaling may still occur in *Myd88-IFNAR* double-KO mice via the TRIF-mediated activation of NF-*κ*B [11] and that complete abrogation of TLR signaling would require the use of *Myd88-Trif* double-KO mice. Despite these limitations, the results from the present study conclusively demonstrate that type I IFNs are not responsible for the preservation of liver regeneration in *Myd88*-deficient mice and make it less likely that TLR signaling is required for liver regeneration.

Cytokine activation after PH modulates the expression of multiple genes involved in liver regeneration, affecting immune, metabolic, growth-related, apoptosis-related, and other signaling networks [22]. As assessed by circulating cytokine levels in the present study, early cytokine activation after PH consists of a marked increase of IL-6 in WT mice, whereas the levels of TNF-*α*, IL-1*β*, IL-12 p70, IL-10, and IFN-*γ* remain below the detection range of these assays in most animals. This observation suggests that the amount of TLR ligands reaching the liver after PH may be much smaller than that of experimental systems involving administration of LPS or pI:pC, which characteristically result in large increases of these cytokines. Consistent with the previously published dependence on MyD88 for triggering cytokine activation after PH, the simultaneous lack of *Myd88* and *IFNAR* is associated with a marked abrogation of the IL-6 increase, without any detectable changes in the levels of other cytokines examined. Importantly, *Myd88-IFNAR* double-KO mice regenerate normally despite the almost complete abrogation of the increase in circulating IL-6. Consistent with what we recently described in *Myd88* KO mice, the initiation of hepatocyte proliferation after PH tended to be accelerated in the double-KO mice. These findings suggest that cytokine activation is not an absolute requirement for liver regeneration, and, together with previous studies, they also suggest that cytokine activation after PH may have both positive and negative effects on hepatocyte proliferation [23–25]. In this regard, the deletion of suppressor-of-cytokine-signaling- (SOCS-) 3 in hepatocytes, a gene that is highly induced in an IL-6 dependent manner, has been shown to confer enhanced proliferative capacity to hepatocytes after PH [25]. The marked attenuation of IL-6 signaling and *Socs3* induction reported in *Myd88* KO mice demonstrated in prior studies [13, 15] could thus be a potential explanation for the preservation of liver regeneration in *Myd88-IFNAR* double-KO mice.

An unexpected finding of the present study was the presence of gross liver lesions in a considerable portion (28%) of *Myd88-IFNAR* double-KO mice. Several studies by other investigators have used *Myd88-IFNAR* double-KO mice to study specific aspects of immune cell activation and host defense against a variety of infectious agents, but no gross physical anomalies have been reported in this mouse strain. It is unclear whether liver lesions passed unnoticed in previous studies, as a systematic assessment of the liver was not formally reported as part of their experimental design [19–21, 26]. Based on the histological appearance of the liver lesions that we found in *Myd88-IFNAR* double-KO mice, the primary etiologic possibilities for these findings are infections, ischemia, and toxins. A toxic origin can be discarded, since no pharmacological agents were administered to the mice other than inhalational isofluorane at the time of the surgery, and the lesions were already present at that point. The distribution of the lesions is atypical for an ischemic injury, and the mice looked healthy without signs of hemodynamic compromise or evidence of ischemia in other organs. We believe that an infection, probably viral, is the most likely explanation for the lesions observed in *Myd88-IFNAR* double-KO mice. Indeed, mice deficient in *IFNAR* have an increased susceptibility to and severity of viral, bacterial, and fungal infections compared to normal mice [27, 28], and this susceptibility is further enhanced in *Myd88-IFNAR* double-KO mice [19, 20]. Nongenetic factors, such as age of acquisition of the infection or different microbial burden, could explain the incomplete penetrance of the liver lesions observed in our study. Further work is needed in order to conclusively determine the cause of the hepatic lesions in the* Myd88-IFNAR* double-KO mice, however.

We conclude that type I IFNs are not responsible for the preservation of liver regeneration seen in *Myd88*-deficient mice. We also suggest that TLR signaling and cytokine activation are not absolute requirements for normal murine liver regeneration. Finally, future studies using *Myd88-IFNAR* double-KO mice should take into account the potential presence of gross liver pathology in these animals, as these lesions could interfere with a diverse range of experimental results.
